# A noncanonical RNA polymerase assembly pathway in *Bacillus subtilis*: α Dimer associates with either β or β′ before forming the core enzyme

**DOI:** 10.1016/j.jbc.2026.111478

**Published:** 2026-04-17

**Authors:** Aniruddha Tewary, Anushka Chakraborty, Runa Sur, Jayanta Mukhopadhyay

**Affiliations:** 1 Department of Chemical Sciences, Bose Institute, UN 80, Sector V, Kolkata, India; 2 Department of Biophysics, Molecular Biology and Bioinformatics, University of Calcutta, Kolkata, India

**Keywords:** RNA polymerase, transcription, *Bacillus subtilis*, protein assembly, bacterial gene regulation

## Abstract

The bacterial RNA polymerase (RNAP) core enzyme, responsible for transcription, is composed of five conserved subunits: α_2_, β, β′, and ω. In *Escherichia coli*, RNAP assembly follows a well-established sequential pathway: α + α → α_2_ → α_2_β → α_2_ββ′(ω). This canonical scheme has long been considered universal. Here, we show that in *Bacillus subtilis*, RNAP assembly proceeds not only *via* the canonical route but also through an alternative pathway: α + α → α_2_ → α_2_β′(ω) → α_2_ββ′(ω). We provide *in vivo* evidence for both α_2_β and α_2_β′ intermediates in *B. subtilis*, whereas *Escherichia coli* supports only the α_2_β intermediate. These findings uncover a previously unrecognized plasticity in bacterial RNAP assembly, attributable to distinct α–β and α–β′ interfaces in different lineages. Our results highlight the evolutionary diversification of RNAP assembly and suggest new opportunities for developing species-specific antibiotics that target lineage-dependent assembly pathways.

Transcription, the first step of gene expression, is catalyzed by RNA polymerase (RNAP), a multisubunit enzyme conserved across all domains of life. In bacteria, the RNAP core enzyme consists of five subunits: two α, one β, one β′, and the small ω subunit. Assembly of these subunits into a functional enzyme is an essential and tightly regulated process, yet much of our current understanding derives from studies of a single bacterial species.

In *Escherichia coli*, which has long served as the model organism for transcription, RNAP assembly has been characterized extensively through *in vitro* reconstitution, cross-linking, and structural studies (1, 2, 3, 4, 5, 6, 7). The prevailing model describes a sequential pathway: two α subunits dimerize to form an α_2_ complex, which recruits the β subunit to generate the α_2_β intermediate. Subsequent addition of β′ yields the catalytically active α_2_ββ′ core, with the ω subunit assisting in β′ folding and stabilizing the complex (8, 9). This stepwise sequence is widely regarded as the canonical model of bacterial RNAP assembly (10, 11, 12).

However, comparative structural and evolutionary analyses have revealed substantial diversity in intersubunit interfaces across bacterial lineages, despite overall conservation of RNAP architecture (13, 14). In *E. coli*, the two α subunits (αI and αII) are functionally asymmetric: αI preferentially engages with β, while αII interacts with β′ (15, 16). A single amino acid substitution at residue R45 of α can bias this interaction, allowing selective binding to β′ but not β (15, 16, 17). Using such mutants, chimeric RNAP complexes have been generated in which WT and mutant α subunits interact separately with β and β′ (15).

This R45 residue is conserved across all bacterial species, including *Bacillus subtilis* (R42). While generating a chimeric *B. subtilis* RNAP with an α42A, we observed that the mutant subunit was able to bind both β and β′ subunits. Strikingly, we observed that the α dimer in *B. subtilis* can associate with either β or β′, forming α_2_β or α_2_β′ intermediates, each of which can subsequently recruit the remaining subunit to generate a functional RNAP core. Both intermediates exist *in vivo* in *B. subtilis*, whereas only α_2_β has been detected in *E. coli*. We also found that independently folded *B. subtilis* β or β′ subunit could bind α_2_β’ or α_2_β, respectively, to form RNAP core enzyme, which is not possible with *E. coli* RNAP assembly.

These findings challenge the long-standing assumption that the *E. coli* pathway represents a universal model of RNAP assembly. Instead, they reveal lineage-specific plasticity in the order of subunit recruitment, suggesting that even fundamental processes such as RNAP assembly can evolve distinct pathways in different bacteria. This divergence has important implications both for understanding transcriptional evolution and for exploiting RNAP assembly pathways as potential species-specific antibiotic targets.

## Results

### β and β′ independently interact with the α dimer to form assembly intermediates

To examine the sequence of RNAP subunit assembly in *B. subtilis*, we monitored the formation of intermediates during *in vitro* reconstitution using the same approach previously applied to *E. coli*. Individual α, β, and β′ subunits were mixed in all possible combinations (α–β, α–β′, β–β′, and α–β–β′) under denaturing conditions, followed by dialysis into reconstitution buffer, purification by affinity chromatography, and analysis by gel electrophoresis.

**Figure 1:**
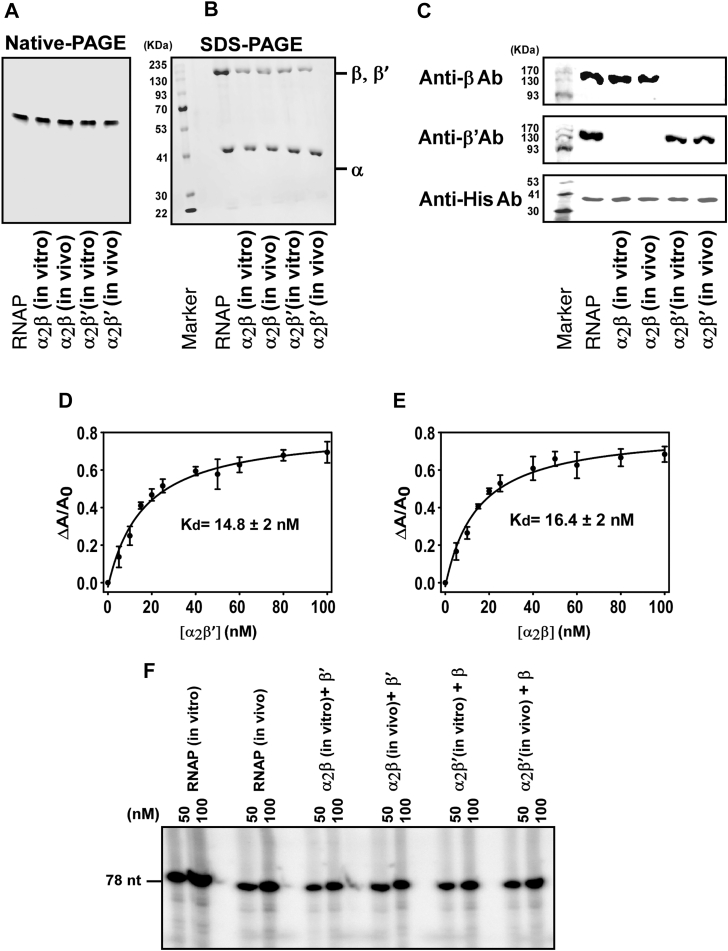
***Bacillus subtilis* β and β′ both independently interact with α subunit to form the assembly intermediate.***A*, His-tagged α was separately reconstituted with either β or β′ *in vitro* and *in vivo*. The purified fractions by Ni-NTA agarose were run in a native PAGE. *B*, the complexes in each band in PAGE were extracted and run in an SDS-PAGE. *C*, three identical SDS-PAGE gels were run as in B and were immunoblotted individually with anti- β, anti- β′, anti- His (detecting α subunit) antibodies. *D*, binding assay of β with α2β′ using fluorescence anisotropy: 10 nM TMR-labeled β was titrated with increasing concentration of α2β’. The fluorescence anisotropy value of β was measured (λex = 540 nm, λem = 580 nm). The experiment was repeated thrice (technical replicates), and the mean values were plotted. The error bars represent standard deviation. Kd was calculated by fitting the data using a single-rectangular hyperbolic function. *E*, binding assay of β′ with α2β using fluorescence anisotropy: Same as A, but 10 nM TMR-labeled β′ was titrated with increasing concentration of α2β. *F*, *in vitro* transcription assay: 50 and 100 nM reconstituted RNAPs were incubated with and 50 nM abrB promoter DNA fragment to form RPo. Transcription was initiated with the addition of NTPs containing αP32 ATP. Run-off transcripts were 78 nt. RNAP, RNA polymerase; TMR, tetramethylrhodamine.

As expected, isolated subunits failed to assemble into RNAP (1). By contrast, simultaneous reconstitution of α, β, and β′ yielded fully assembled RNAP. Consistent with earlier studies in *E. coli*, α readily formed a complex with β (α–β) (1, 2, 3, 4, 5, 6, 7) (Fig. 1, *A* and *B*). Strikingly, however, α also formed a stable complex with β′ (α-β ′) (Fig. 1, *A* and *B*), an interaction not observed in *E. coli*. No interaction was detected between β and β′ in the absence of α (Fig. S1*A*).

To validate these findings *in vivo*, all possible combinations of RNAP subunits were co-expressed in *E. coli*, followed by affinity purification with His-tagged α. The same intermediates were recovered (α–β and α–β′), confirming the *in vitro* results (Fig. 1, *A* and *B*). Western blotting verified the identity of the purified subunits (Fig. 1*C*). Note that *B. subtilis* α, when expressed in *E. coli,* does not associate with β or β′ subunits from the host cell, consistent with earlier reports that recombinant expression does not interfere with endogenous RNAP assembly (18) (Fig. S1*B*). In addition, *in vitro* reconstitution assays performed using His-tagged *B. subtilis* α along with *E. coli* β and β′ subunits, followed by Ni-NTA affinity purification, showed that neither β nor β′ co-eluted with α (Fig. S1*C*).

### Both α_2_β and α_2_β′ intermediates assemble into catalytically active RNAP

To test whether α–β and α–β′ complexes could recruit the missing subunit and form complete RNAP, we performed fluorescence anisotropy–based binding assays. Tetramethylrhodamine (TMR)-labeled β and β′ were titrated with α–β′ and α–β complexes, respectively. In both cases, binding occurred at nearly equimolar stoichiometry (Fig. 1, *D* and *E*).

Subsequent *in vitro* transcription assays confirmed that addition of the missing subunit produced fully functional RNAP holoenzymes (Figs. 1*F* and S2). Importantly, these complexes contained α_2_β and α_2_β′ dimers rather than simple αβ or αβ′ assemblies (Fig. 1). Together, these results demonstrate that *B. subtilis* supports two distinct intermediates—α_2_β and α_2_β′—each capable of maturing into transcriptionally active RNAP.

### Both assembly pathways operate *in vivo*

**Figure 2:**
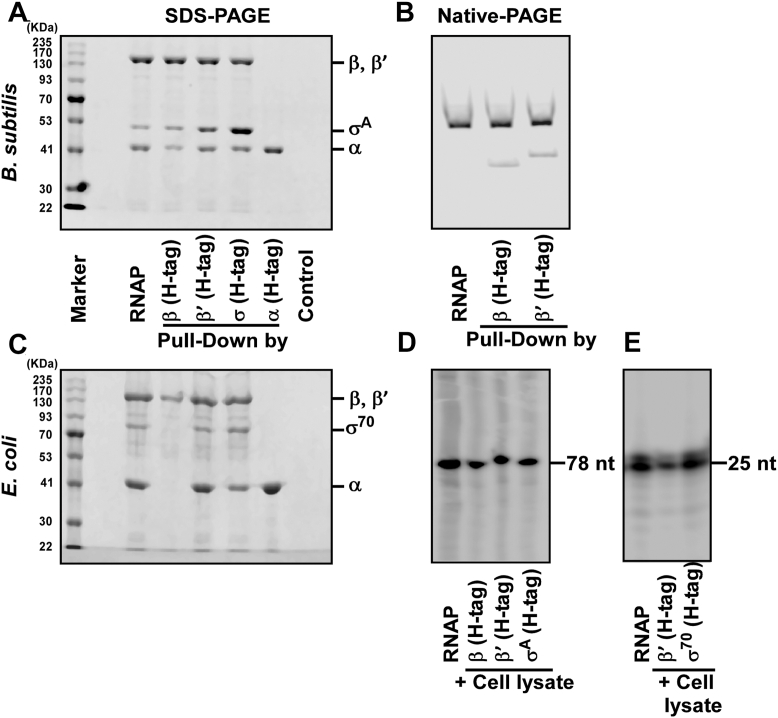
**Both assembly pathways are simultaneously present *in vivo* in *Bacillus subtilis*.***A*, pull-down assay: protein extract from *Bs168*cells was divided equally into five parts and was incubated with 20 μl 2.5 μM His tagged- α, His tagged- β, His tagged- β′, His tagged- σ^A^, and no protein. The samples were purified by Ni-NTA, run on an SDS-PAGE, and stained by Coomassie blue. *B*, same as A, but β and β′ pulled down fraction was run in a native PAGE. *C*, same as A, but *Escherichia coli* MG1665 cells and His-tagged *Ec* RNAP subunits were used. *D*, *in vitro* transcription assay, 100 nM pulled down RNAP samples and 50 nM *abrB* promoter DNA fragment were incubated to form RPo. Transcription was performed as in Fig. 1F. *E*, same as *D*, but *Ec* RNAP, subunits, and *lacUV5* promoter DNA fragment. RNAP, RNA polymerase.

We next asked whether both intermediates exist *in vivo*. Pull-down assays were performed from *B. subtilis* extracts using His-tagged β or β′. In both cases, RNAP was successfully recovered and shown to be transcriptionally active (Figs. 2, *A*, *B*, and *D* and S2). By contrast, His-tagged α failed to pull down RNAP, consistent with its role as an obligate dimer (Fig. 2, *A* and *B*). Interestingly, RNAP complexes purified with His-tagged β or β′ were associated with σA, likely reflecting the free σ factor present in the cell (19, 20, 21). However, binding assays showed that σA does not interact directly with α_2_β or α_2_β′ intermediates (Fig. S1, *D* and *E*). Control experiments with purified intein-chitin-binding domain (CBD)–tagged RNAP cores excluded nonspecific interactions of β or β′ with assembled RNAP (Fig. S3).

Thus, the results confirm that both α_2_β and α_2_β′ intermediates exist *in vivo* in *B. subtilis*. In contrast, only α_2_β was detected in *E. coli*, consistent with previous observations (1, 2, 3, 4, 5, 6, 7) (Fig. 2, *C* and *E*).

### Role of the ω subunit in assembly

**Figure 3:**
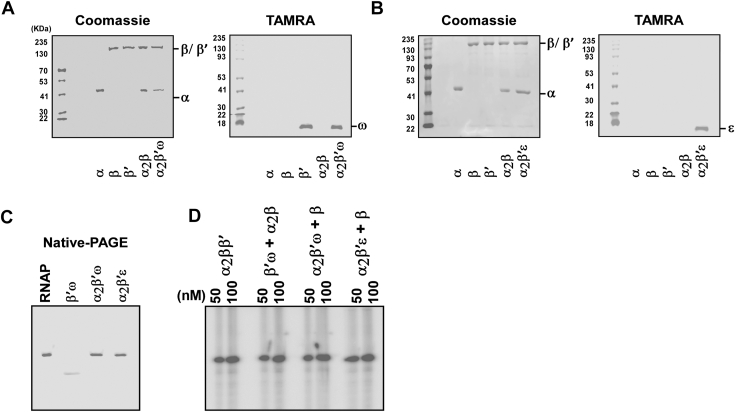
**Association of ω and ε subunit in RNAP assembly.***A*, TAMRA-labeled ω subunit was reconstituted along with α-His, β-His, β′-His, α_2_(-His)β, α_2_(-His)β′, purified by Ni-NTA affinity chromatography and run on an SDS-PAGE. The PAGE was scanned by fluorescence imaging (*right panel*) followed by staining with Coomassie blue (*left panel*). For the *right panel*, prestained Marker and labeled protein was detected with different wavelength scan and merged into a single image by using Bio-Rad Image Lab Software. *B*, same as A, but with TAMRA-labeled ε. *C*, α_2_ββ'ω (RNAP), β′ω, α_2_β'ω, α_2_β'ε (ω and ε was labeled with TAMRA) were run in a native PAGE and scanned with fluorescence imaging. *D*, β′ω, α_2_β'ω, α_2_β'ε were incubated with α_2_β, β, and β respectively at 37 °C for 15 min. *In vitro* transcription assays were performed as in 1F. RNAP, RNA polymerase; TAMRA, tetramethylrhodamine

The ω subunit has previously been shown to assist in *E. coli* RNAP assembly, though it is not essential for catalytic activity (1, 2, 3, 4, 5, 6, 7, 8, 9). Similarly, ω-free *B. subtilis* RNAP remains transcriptionally active (18). To test its role, we reconstituted α, β, β′, and α_2_β/α_2_β′ complexes in the presence of carboxytetramethylrhodamine (TAMRA)-labeled ω. Electrophoretic mobility shift assays revealed that ω associated with β′ and α_2_β′, but not with β or α_2_β (Fig. 3, *A* and *C*). The addition of the missing subunit completed assembly into active RNAP (Fig. 3*D*). Deletion of the *rpoz* gene (encoding ω subunit) from the genome of *B. subtilis* does not affect the formation of either α_2_β and α_2_β′ intermediates or RNAP assembly (Fig. S4, *A* and *B*). However, there is a ∼20% decrease in the transcription efficiency of RNAP purified from the *Δrpoz* strain compared to WT (Fig. S4*C*). These findings suggest that, as in *E. coli*, ω contributes to stabilizing β′ at the intermediate stage but is not strictly required for RNAP function.

### Role of the ε subunit in assembly

Firmicutes RNAPs, including *B. subtilis*, contain an additional subunit, ε, implicated in phage defense (22). Using reconstitution assays, we observed that ε bound specifically to α_2_β′, but not to α, β′, or α_2_β (Fig. 3, *B* and *C*). This interaction is consistent with structural data showing that ε contacts both α and β′ in *B. subtilis* RNAP (23). The resulting α_2_β′ε complex readily incorporated β to form α_2_ββ′ε (Fig. 3*D*).

Importantly, the presence of ε did not affect transcriptional activity, suggesting that its role lies outside catalysis, possibly in RNAP stability or phage resistance.

### Structural basis of divergence in RNAP assembly

**Figure 4:**
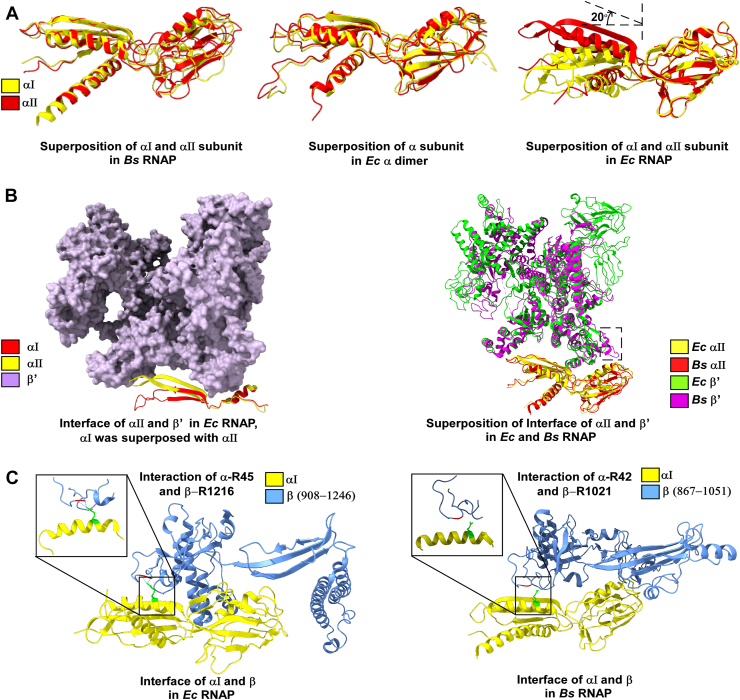
**Structural analysis of the interface of α with β and β′ in *Bacillus subtilis* and *Escherichia coli****. A*, *left panel*: superposition of αI subunit with αII in *B. subtilis* (RMSD, 4.2 Å); *middle panel*: superposition of one α subunit with another in *Ec* α dimer (RMSD, 1.91 Å); *right panel*: superposition of αI subunit with αII in *E. coli*, ∼20° angular shift of the β-sheet spanning residues 180 to 207 and α-helix spanning residues 34 to 50, pivoting around Val180 is shown (RMSD, 5.64 Å). *B*, *left panel*: subunit interface αII with β′ in *E. coli*; *right panel*: superposition of αII-β′ interface of *E. coli* and *B. subtilis*, extra helix in *B. subtilis* is shown in the *dotted rectangle*. *C*, *left panel*: subunit interface αI with β in *E. coli*, only part of β is shown, interaction between R45 of αI with R1216 and G1215 of β is highlighted; *right panel*: subunit interface αI with β in *B. subtilis*, R42 of αI does not interact with R1021 of β.

The ability of *B. subtilis* α_2_ to bind either β or β′ reflects fundamental differences in subunit interface architecture compared to *E. coli*. Structural analyses show that the α dimer in *B. subtilis* is more symmetric (23), enabling equivalent interactions with β or β′ (Fig. 4*A*). The architectures of the two *Ec* α subunits remain symmetric in the dimer without any interaction with β or β′ (24). However, the structural and mutational analyses of *Ec* RNAP have displayed an asymmetry in the architecture and function between the two α subunits, with one preferentially engaging with β and the other with β′ (15, 17, 25) (Fig. 4*A*). The β′-binding groove of *Ec* αII exhibits an angular constriction compared to αI. Thus, the binding of β′ to a dimer induces a rearrangement of several structural elements. The region comprising residues 186 to 201 (17, 26, 27, 28) undergoes a transition into a more extended and defined antiparallel β-sheet, now encompassing residues 180 to 192 and 195 to 207. This is further accompanied by an ∼20° angular shift of the structural elements comprising β-sheet (residues 180–207) and an α helix (residues 34–50), pivoting around Val180, effectively narrowing the groove to bring the interaction surfaces of αII in the proximity to β′ (Fig. 4*B*). This structural transition is likely to accommodate the β′ into the binding groove of α, and stabilize the α–β′ interactions and represent a key step in the assembly process of RNAP. The critical role of α residue R45 in *E. coli* further illustrates lineage-specific determinants. R45 lies within hydrogen-bonding distance of β residues G1215 and R1216, stabilizing α–β interactions (29). In *B. subtilis*, the corresponding residue (R42) is separated by nearly 7 Å from β-Arg1021 and cannot form hydrogen bonds (Figs. 4*C* and S5). In addition, the β-HIN region, essential for RNAP assembly in *E. coli* (26, 30), contains two long helices that are absent in *B. subtilis*, where the HIN region is shorter. Conversely, *B. subtilis* β′ carries an additional α-helix absent in *E. coli*, located near residue 80 of αII, likely providing an alternative interaction site (Fig. 4*B*). Together, these structural differences explain why *B. subtilis* permits parallel α_2_β and α_2_β′ intermediates, whereas *E. coli* restricts assembly to the α_2_β pathway.

## Discussion

The canonical model of bacterial RNAP assembly, derived from *E. coli*, proposes that α_2_ first recruits β to form the α_2_β intermediate, which then incorporates β′ to yield the core enzyme (1, 2, 3, 4, 5, 6, 7). Our findings in *B. subtilis* challenge this paradigm by demonstrating that α_2_ can associate with either β or β′, forming two alternative intermediates—α_2_β and α_2_β′—both capable of producing catalytically active RNAP. This establishes that the order of subunit addition is not universally conserved but instead varies across bacterial lineages.

These findings highlight that RNAP assembly has evolved distinct solutions in different bacterial lineages. The parallel assembly pathways in *B. subtilis* may enhance the robustness and efficiency of RNAP biogenesis, ensuring enzyme availability under diverse physiological conditions. More broadly, they demonstrate that even highly conserved cellular machines can accommodate lineage-specific flexibility at the level of assembly.

Because RNAP is a central and essential enzyme, its assembly has long been considered a potential drug target. Our results suggest that subunit interfaces differ significantly across bacterial groups, raising the possibility of designing species-specific inhibitors that selectively disrupt RNAP assembly in pathogens without affecting commensal bacteria.

## Experimental procedures

### Cloning of *rpoA, rpoB,* and *rpoC* genes

The oligonucleotide primers used for cloning are listed in Table S1.

*B. subtilis rpoA, rpoB,* and *rpoC* genes were amplified from pNG219 (18) and individually cloned in pCOLADuet-1 vector using BamHI-KpnI, pACYCDuet-1 vector using BamHI-KpnI, and NcoI-KpnI, and pCDFDuet-1 vector using NdeI-KpnI. To incorporate an intein CBD at the C terminus of *Bs rpoB* gene in plasmid pNG545 (18), a stop codon at the end of *rpoB* gene was removed by site-directed mutagenesis and *MxeGyrA intein* genes were amplified by PCR from plasmid pTWIN1 using oligonucleotide primers (Table S1) and cloned in mutated pNG545 using KpnI-XhoI. An adenine base was inserted just after KpnI sequence to make intein-CBD in frame.

### Site-directed mutagenesis of *rpoA, rpoC*, and *sigA* genes

Subsequently, 42nd codon of the *rpoA* gene located in the plasmid pCOLADuet-1 was mutated to introduce an alanine codon by site-directed mutagenesis to generate pCOLADuet-1 *rpoA* R42A.

A stop codon was inserted immediately after the *rpoC* gene in pNG540 to generate pNG540M.

Single cysteine derivatives of σ^A^ were generated by site-directed mutagenesis in pET28a-*sigA* (31).

### Generation of *rpoZ* KO strain

A DNA fragment containing the *AmpR* gene along with its promoter was amplified from the pDG148, such that it was flanked on both sides by ∼500 bp regions homologous to sequences upstream of the *rpoZ* start codon and downstream of the *rpoZ* stop codon, using oligonucleotide primers (Table S1) as in (32). Electroporation of the DNA in *Bs168*cells was performed as in (33).

### Purification of RNAP

For purification of RNAP (α-His), B834(DE3) cells were transformed with pCOLADuet-1-*rpoA*, pACYCDuet-1-*rpoB*, and pNG540M. Induction and purification were done as in (34).

Purification of RNAP (β′-His) was performed as in (34).

For purification of RNAP (without His-tag) the plasmids pNG540M and pNG545 β-intein CBD were cotransformed and expressed in B384. The protein was purified as in (35).

### Purification of σ^A^

Purification of σ^A^ was performed as in (34).

### Purification of RNAP subunits

For purification of α and αR42A, B834(DE3) cells were transformed with pCOLADuet-1-*rpoA* and pCOLADuet-1-*rpoA* R42A, respectively. Transformed cells were grown in LB media till the absorbance reached 0.5 and the cells were induced with 0.5 mM IPTG at 16 °C for 14 h. Cells were harvested by centrifugation, resuspended in lysis buffer (50 mM Tris–Cl, 5% glycerol, 200 mM NaCl, 5 mM β-ME, and 1 mM PMSF) and were disrupted by sonication. The lysates were spun at 16,000*g* for 15 min at 4 °C. The supernatant was loaded onto Ni-NTA column (Bio-Rad) pre-equilibrated with Tris-glycerol (TG) buffer (50 mM Tris–Cl, 5% glycerol, and 500 mM NaCl). α was eluted using a step gradient of imidazole in TG.

For purification of β and β-His, B834 cells were transformed with pACYCDuet-1-*rpoB* and pACYCDuet-1-*rpoB-His,* respectively. Induction and purification were done as in (35).

For purification of β′ and β′-His, B834(DE3) cells were transformed with pCDFDuet-1-*rpoC* and pNG540, respectively. Induction and purification were done as in (35).

Notably, the pNG540 expresses both β′-His and ω. While purifying β′-His by Ni-NTA, ω was eluted as the unbound fraction. The samples containing ω was further purified by size-exclusion chromatography using Bio-Gel P30.

For purification of ε subunit, B834 (DE3) cells were transformed with pNG567 (18) and the steps as in the purification of ω were followed.

### Reconstitution of RNA polymerase and intermediates

The purified α subunit was precipitated by the addition of 0.4 g/ml ammonium sulfate, centrifuged at 18,000*g* for 30 min at 4 °C. The pellet was dissolved in buffer A (6M guanidine hydrochloride, 50 mM Tris–Cl pH 7.9, 10 μM ZnCl_2_, 10 mM MgCl_2_, 1 mM EDTA, 20 mM β-ME, 1 mM PMSF, and 10% glycerol).

α, β, β′ in buffer A were mixed in different combination: (i) α-(His) and β at a molar ratio of 1:2; (ii), α-(His) and β′ at a molar ratio of 1:2; (iii), α-(His), β, and β′ at a molar ratio of 1:2:2. Notably, higher molar concentration of β and β′ was used as a significant fraction of β and β′ tends to precipitate during the dialysis. Reconstitution and purification were done as in (35).

### Assembly of subunits in a recombinant overexpression system

B834 cells were cotransformed with the following sets of plasmids; (i) pCOLADuet-1-*rpoA* and pACYCDuet-1-*rpoB*, (ii) pCOLADuet-1-*rpoA* and pCDFDuet-1-*rpoC*, (iii) pCOLADuet-1-*rpoA*, pACYCDuet-1-*rpoB*, and pCDFDuet-1-*rpoC.* Transformed cells were grown in LB supplemented with 0.1% dextrose and respective antibiotics at 37 °C till the absorbance reached 0.5. Induction, cell lysis, and affinity chromatography was done as in the purification of α. The purified samples were run on the native PAGE (Fig. 1*A*) and SDS PAGE (Fig. S6).

### Labeling of proteins

σ^A^ (124c), β and β′ were labeled with Tetramethyl rhodamine-6-maleimide following the process as in (34).

ω and ε were labeled with TAMRA-NHS ester following manufacturer’s protocol, except the labeling time was reduced to 20 min to avoid labeling of all the lysine residues, which may interfere its binding to RNAP.

### Purification of *Ec* α, β, β,′ and σ70

For purification of α, β, β′, and σ70, BL21 cells were transformed with pREII-NHα, pRL706, pRL663, and pGEMD-σ70. α, β, and β′ were purified as in the purification of those subunits of *B*. *subtilis.* σ70 was purified as in (35).

### Preparation of promoter DNA fragments

The linear DNA promoter fragments (*abrB* and *lacUV5*) were generated by PCR amplification using oligonucleotides (Table S1).

### Fluorescence anisotropy measurement

Briefly, 10 nM TMR-labeled β was titrated with increasing concentration of α_2_β′ at 37 °C and the anisotropy values were measured (λ_ex_ = 540 nm, λ_em_ = 580 nm) using a PTI Fluorescence Master QM400 system fitted with an automatic polarizer.

Normalized fluorescence anisotropy values (Δ*A*/*A*o, where Ao is the anisotropy value of β and A is the anisotropy value of α_2_β′ bound β) were plotted against the α_2_β' concentration using the SigmaPlot software (Systat Software Inc). The dissociation constants (*K*_*d*_) of the binding for β and α_2_β' was determined by fitting the data to three-parameter single rectangular hyperbolic functions.

Subsequently, 10 nM TMR-labeled β′ was titrated with increasing concentration of α_2_β, and K_d_ of the binding was determined by fluorescence anisotropy as mentioned above.

### *In vitro* transcription assay

α_2_β and α_2_β′ were mixed with β′ and β, respectively, in 1:2 M ratio in transcription buffer (18 mM Tris (pH 8.0), 10 mM NaCl, 8 mM β-ME, and 10 mM MgCl_2_) for 15 min at 37 °C to form RNAP core. RNAP core was incubated with 2-fold concentration of σ at 25 °C for 15 min to form holo enzyme. In addition, 50 nM promoter *abrB* DNA fragment was mixed with 50 nM RNAP in transcription buffer and incubated at 37 °C for 15 min to form the open complex (RPo). Transcription was initiated with NTP (final concentrations: 250 μM of UTP, GTP, CTP, and 25 μM of [α-32 P] ATP) supplemented with 25 ng/μl heparin, and the reaction was kept at 37 °C for 15 min. The reaction was stopped by the addition of formamide loading buffer (FLB) (80% formamide, 10 mM EDTA, 0.01% bromophenol blue, and 0.01% xylene cyanol) followed by heating at 95 °C for 5 min. The reaction mixtures were resolved in 12% urea-PAGE and scanned by a storage phosphor scanner (Amersham Typhoon, GE Healthcare).

### Western blot assay

Equal amounts of RNAP (α-His), α_2_β, and α_2_β' were run in 10% SDS-PAGE, followed by western blot using anti-His, anti-β, and anti-β′ antibodies (BioBharati Lifescience) as in (36). Notably, the anti-β and anti-β′ antibodies were raised against the *Bs* β and β′ subunits, yet they also detect the corresponding *Ec* subunits.

### *In vivo* pull-down assay

*Bs168*cells were grown in 5L LB media at 37 °C till the absorbance reached 0.8. Cells were harvested and lysed as in the purification of α. The supernatant was precipitated by the addition of 0.4 g/ml ammonium sulfate, centrifuged at 18,000*g* for 30 min at 4 °C. The pellet was dissolved in Buffer B (50 mM Tris–Cl pH 7.9, 50 mM KCl, 10 μM ZnCl_2_, 10 mM MgCl_2_, 1 mM EDTA, 5 mM β-ME, 1 mM PMSF, and 20% glycerol) and divided into five tubes. Briefly, 20 μl 2.5 μM α-His, β-His, β′-His, and σ^A^-His were added separately to the four fractions, and the last fraction was kept as a control. The reaction mixture was incubated at 37 °C for 15 min and then loaded onto a Ni-NTA column pre-equilibrated with TG. The column was subsequently washed with 40 mM imidazole in TG. The proteins were eluted using a step gradient of imidazole in TG. The eluted samples were concentrated and subjected to SDS-PAGE analysis.

To test whether the same β or β′ used in pull-down assay were incorporated in the RNAP, same assay was performed with TMR-labeled β-His and TMR-labeled β′-His. The concentrated samples were run in a native PAGE.

As a control, *E. coli* MG1665 cells were grown, and an *in vivo* pull-down assay was performed with *Ec* His-tagged subunits as above.

### Analyzing protein interaction interfaces

We employed UCSF chimera’s (37) Matchmaker tool to superimpose protein structures (Protein Data Bank (PDB): 6CZA, 1BDF, and 6C9Y) (23–25) and utilized the Distance tool to measure interatomic distances for structural comparison and analysis.

## Data availability

Data will be made available on request.

## Supporting information

This article contains supporting information.

## Conflict of interest

The authors declare that they have no conflicts of interest with the contents of this article.
