# Supplementary material for: A noncanonical RNA polymerase assembly pathway in Bacillus subtilis: α Dimer associates with either β or β′ before forming the core enzyme
Source: J Biol Chem. 2026 Apr 17;302(6):111478. doi: 10.1016/j.jbc.2026.111478 (PMC13202282; doi:10.1016/j.jbc.2026.111478)
Supplement: Supporting information [file mmc1.docx]

**Supporting Information for:**

**A noncanonical RNA polymerase assembly pathway in *B. subtilis*: α dimer associates with either β or β′ before forming the core enzyme**

Authors: Aniruddha Tewary^1^, Anushka Chakraborty^1,2^, Runa Sur^2^, and Jayanta Mukhopadhyay^1^


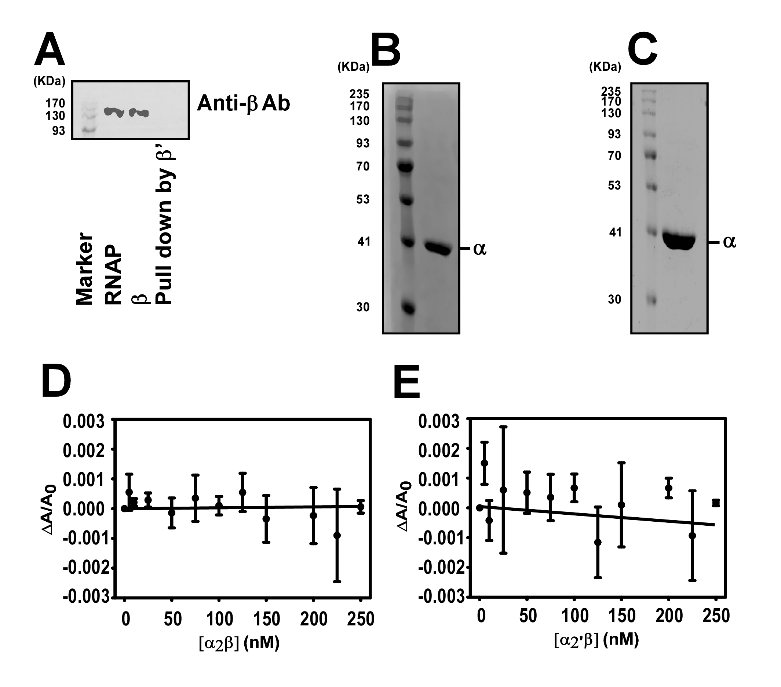
**Figures:**

**Fig. S1: β and β’ does not interact in absence of α subunit and σ^A^ does not bind to the assembly intermediate.**

A, His-tagged β' was reconstituted with β *in vitro* followed by Ni-NTA affinity chromatography. The sample was run in an SDS-PAGE and immunoblotted with anti- β antibody.

B, *Bs* His-tagged α was overexpressed in BL21 (DE3)*.* The cells were lysed, and the clarified supernatant was subjected to Ni-NTA affinity chromatography. The sample was run in an SDS-PAGE.

C, *Bs* His-tagged α was reconstituted with *Ec* β and β' *in vitro* followed by Ni-NTA affinity chromatography. The sample was run in an SDS-PAGE.

D, 10 nM TMR-labelled σ^A^ was titrated with increasing concentration of α_2_β complex. The fluorescence anisotropy value of σ^A^ was measured with excitation at 540 nm and emission at 580 nm. The experiment was repeated thrice (technical replicates) and the mean values with standard deviations were plotted.


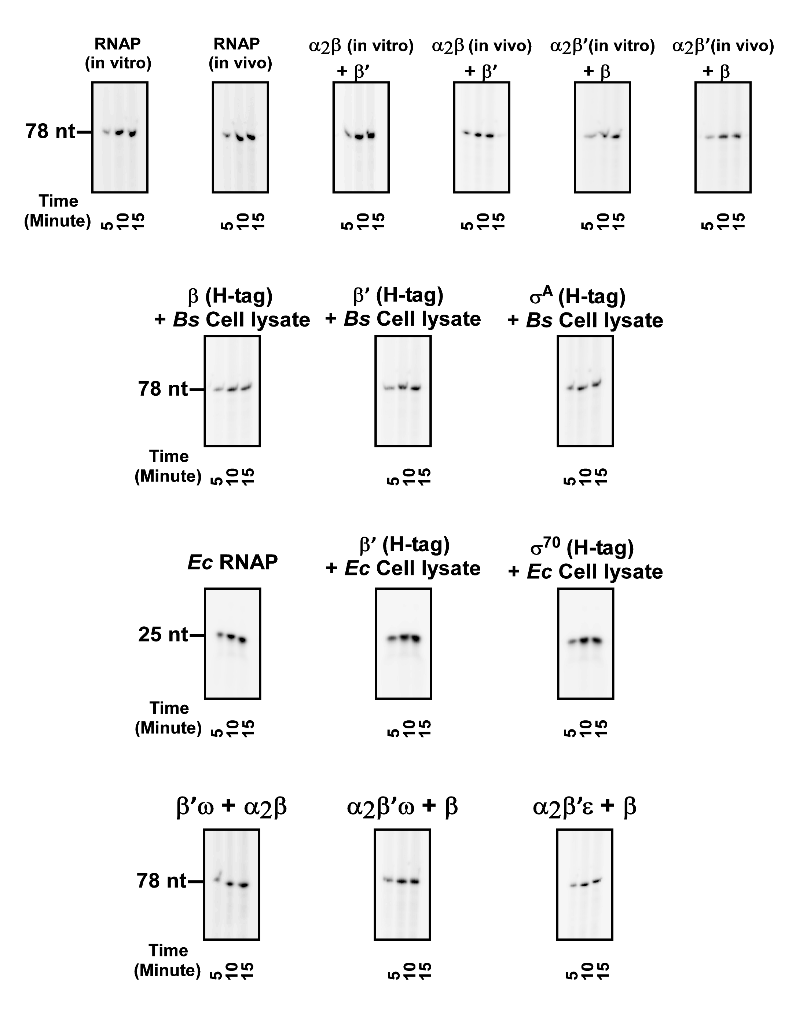
E, Same as D, except titrated with α_2_β' complex.

**Fig. S2: In-vitro transcription assay using time points**

100 nM reconstituted RNAPs containing 200 nM σ^A^ were incubated with 50 nM promoter DNA fragment to form RPo. Transcription was initiated with the addition of NTPs containing α^P32^ ATP and reactions were incubated for different time points.


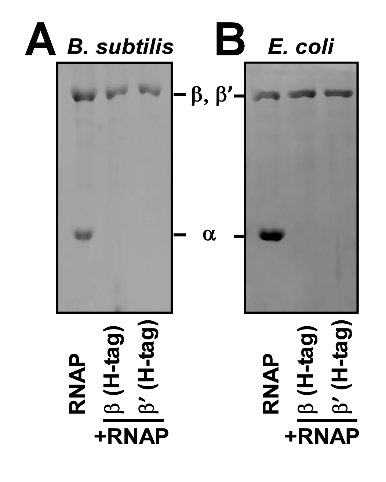


**Fig. S3: β and β’ does not bind to the RNAP core enzyme non-specifically.**

A, *Bs* RNAP samples were mixed with His-tagged-β and His-tagged β' separately, incubated at 37^o^C for 15 minutes, followed by Ni-NTA affinity chromatography. Intein-CBD tagged RNAP was used. The eluted samples were run in an SDS-PAGE.

B, Same as A, but *Ec* RNAP and subunits were used.


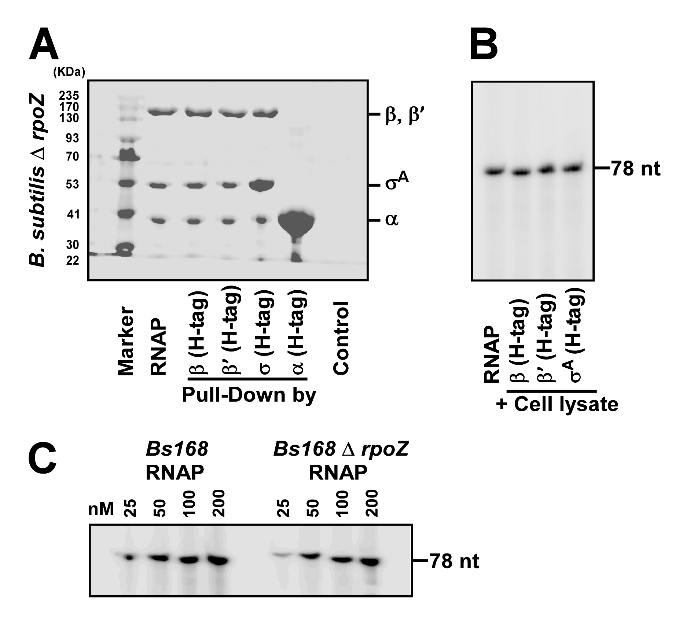


**Fig. S4: Deletion of the *rpoz* gene does not affect the formation of either α₂β and α₂β′ intermediates.**

A, Pull-down assay: protein extract from *Bs168 ΔrpoZ* cells was divided equally into 5 parts and was incubated with 20 μl 2.5 μM His tagged- α, His tagged- β, His tagged- β’, His tagged- σ^A^ and no protein. The samples were purified by Ni-NTA, run on an SDS-PAGE, and stained with Coomassie blue.

B, In-vitro transcription assay, 100 nM pulled down RNAP samples and 50 nM *abrB* promoter DNA fragment were incubated to form RPo. Transcription was performed as in Fig. 1F.

C, In-vitro transcription assay, 25-200 nM RNAP samples from *Bs168* and *Bs168 ΔrpoZ* cells and 50 nM *abrB* promoter DNA fragment were incubated to form RPo. Transcription was performed as in Fig. 1F.


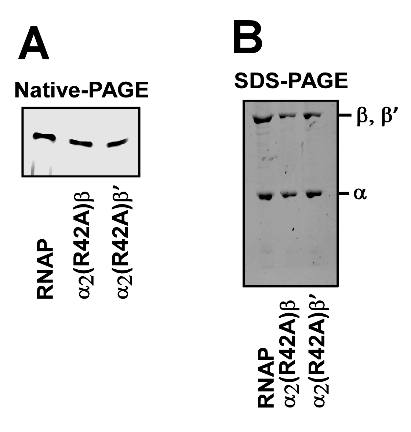


**Fig. S5: 42^th^ Arginine residue of *Bs* α subunit is not crucial for β binding.**

A, His-tagged α R42A was separately reconstituted with either β or β’ *in vitro*. The purified fractions by Ni-NTA agarose were run in a native PAGE.

B, the protein samples in native PAGE, as in A, were extracted and were run in an SDS-PAGE


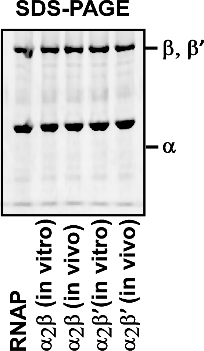


**Fig. S6:** ***Bs* β and β’ both independently interact with α subunit to form the assembly intermediate.**

*Bs* His-tagged α was separately reconstituted with either β or β’ *in vitro*, and *in vivo* by a recombinant expression system in *E. coli*. The purified fractions by Ni-NTA agarose were run in an SDS-PAGE.

**Table. S1:** Sequence of oligonucleotide (5’ - 3’) used for amplification of DNA by PCR

| *rpoA* BamHI fw | AAGGATCCAATGATCGAGATTGAAAAACCAAAAATCGAAACG |
| --- | --- |
| *rpoA* KpnI rev | ACGCGGTACCTCAATCGTCTTTGCGAAGTCCGAG |
| *rpoB* BamHI fw | AAGGATCCAATGACAGGTCAACTAGTTCAGTATGG |
| *rpoB* KpnI rev | TATGAAGGTACCTTATTCTTTTGTTACTACATCGCGTTCAAC |
| *rpoB* NcoI fw | AATGCACCATGGATGACAGGTCAACTAGTTCAGTATGG |
| *rpoC* NdeI fw | CTCATTCATATGTTGCTAGATGTGAACAATTTTGAGTATATGAAC |
| *rpoC* KpnI rev | ATCGATGGTACCTTATTCAACCGGGACC |
| *rpoB* stop fw | GTAACAAAAGAATATTTCTCTACTTATAGCG |
| *rpoB* stop rev | CGCTATAAGTAGAGAAATATTCTTTTGTTAC |
| MxeGreA Intein fw | ATTGGTACCATGCATCACGGGAGATGCACTAGTTGCC |
| CBD rev | AAACTCGAGTCATTGAAGCTGCCACAAGG |
| *rpoA* R42A fw | TCCTTACGTGCTATCCTCTTATCC |
| *rpoA* R42A rev | GGATAAGAGGATAGCACGTAAGGA |
| *rpoC* stop fw | GAAGGTACCTGACATCACCATCAC |
| *rpoC* stop rev | GTGATGGTGATGTCAGGTACCTTC |
| *sigA* A124Cfw | GAAGAAATCGCCTACTGTCAAAAGATTGAAGAAGGT |
| *sigA* A124Crev | ACCTTCTTCAATCTTTTGACAGTAGGCGATTTCTTC |
| *abrB* -55 Fw | ATCAATAGTAAACAAAATGATTG |
| *abrB* +78 rev | CTTGGGAGGAGAATGTTTATG |
| *lacUV5* -51 fw | TAGGCACCCCAGGGCTTT |
| *lacUV5* +25 rev | GTGAAATTGGTATCCCCTCACAATTCCAC |
| DEL *RPOZ* UP FW | ATATTCGATTTCAGTAACCACAAGAAGTCCAAGAGAGG |
| DEL *RPOZ* UP REV- | GAATCAATTGACGGATCTAACATTGATTATTCAACCTCCA |
| DEL *RPOZ* UP REV PDG148 FUSION | GAATCAATTGACGGATCTAACATTGATTATTCAACCTCCATATTTTTCTAAATACATTCAAATAT |
| AMP CASETTE FW | ATATTTGAATGTATTTAGAAAAATAAACAAATAGGGGTTCCGCG |
| AMP CASETTE REV | GAGATAGGTGCCTCACTGATTAAGCATTGGTAA |
| DEL *RPOZ* DOWN FW | GCAGAGGGGGAGAATTCATTGCTTAACAATC |
| DEL *RPOZ* DOWN REV | ATCAGCGATGCCGTTTGCCAGTTTTC |
| DEL *RPOZ* DOWN FW PDG148 FUSION | AAAATAAACAAATAGGGGTTCCGCGGCAGAGGGGGAGAATTCATTGCTTAACAATC |
